# A Novel Structural Framework for α_1A/D_-Adrenoceptor Selective Antagonists Identified Using Subtype Selective Pharmacophores

**DOI:** 10.1371/journal.pone.0019695

**Published:** 2011-05-10

**Authors:** Emily S. Stoddart, Sevvandi Senadheera, Iain J. A. MacDougall, Renate Griffith, Angela M. Finch

**Affiliations:** 1 Department of Pharmacology, School of Medical Sciences, University of New South Wales, Kensington, New South Wales, Australia; 2 School of Environmental and Life Sciences, The University of Newcastle, Ourimbah, New South Wales, Australia; Kings College, London, United Kingdom

## Abstract

In this study four and five-feature pharmacophores for selective antagonists at each of the three α_1_-adrenoceptor (AR) subtypes were used to identify novel α_1_-AR subtype selective compounds in the National Cancer Institute and Tripos LeadQuest databases. 12 compounds were selected, based on diversity of structure, predicted high affinity and selectivity at the α_1D_- subtype compared to α_1A_- and α_1B_-ARs. 9 out of 12 of the tested compounds displayed affinity at the α_1A_ and α_1D_ -AR subtypes and 6 displayed affinity at all three α_1_-AR subtypes, no α_1B_-AR selective compounds were identified. 8 of the 9 compounds with α_1_-AR affinity were antagonists and one compound displayed partial agonist characteristics. This virtual screening has successfully identified an α_1A/D_-AR selective antagonist, with low µM affinity with a novel structural scaffold of a an isoquinoline fused three-ring system and good lead-like qualities ideal for further drug development.

## Introduction

The α_1_-adrenoceptors (ARs) are members of the G-protein-coupled receptor (GPCR) superfamily, which share a conserved structure of seven transmembrane helices [1]. To date, three different α_1_-AR subtypes, namely α_1A_-, α_1B_- and α_1D_-ARs, have been cloned and characterized [2]. Like other ARs, the α_1_-ARs mediate the actions of the endogenous catecholamines norepinephrine and epinephrine [2]. The α_1_-ARs' primary function is in the contraction of smooth muscle in blood vessels, lower urinary tract and prostate [3], [4]. The α_1_-ARs also have functional roles in the central nervous system [5], [6], [7] and the heart [8]. However, assigning distinct physiological roles to the α_1_-AR subtypes has been restricted by the absence of highly potent and selective ligands devoid of ancillary pharmacology. Ligands that can discriminate between α_1_-AR subtypes based on 100–1000 fold differences in affinity would therefore greatly enhance the task of establishing the distribution and physiological functions of the individual α_1_-AR subtypes.

The specificity of α_1_-AR subtype selective antagonists towards their intended target is a crucial issue due to similarity in receptor structure within the biogenic amine GPCRs [9]. Consequently, many non-subtype selective α_1_-AR antagonist therapeutics used for the treatment of hypertension and benign prostatic hypertension (BPH) are associated with side effects such as increased incidence of heart failure, orthostatic hypotension, erectile dysfunction and dizziness [10]. The development of ligands with enhanced subtype selectivity therefore holds promise for therapeutics with decreased incidence of side effects.

Although the α_1A_-AR is the most commonly targeted subtype for the treatment of BPH, the α_1D_-AR may also be considered a suitable target for both BPH and hypertension. α_1D_-AR knockout mice display a reduction of systolic and arterial blood pressure [11] and increased blood pressure following dietary salt-loading [12], [13]. The α_1D_-AR is also the predominant subtype in the human bladder detrusor [14] and pharmacological studies using the α_1D_-AR selective antagonist BMY7378 and the selective α_1A/D_-AR drug tamsulosin indicate α_1D_-AR blockade improves the lower urinary tract symptoms of BPH [15]. A selective α_1D_-AR antagonist may thus have therapeutic potential for the treatment of hypertension and BPH. It is therefore the aim of our research to identify a structurally novel antagonist characterized by high affinity and selectivity towards the α_1D_-AR compared to α_1A_- and α_1B_-ARs, and other biogenic amine GPCRs.

Recently we reported four and five-feature antagonist pharmacophores for the α_1A_, α_1B_ and α_1D_ -ARs, that were developed using training sets of subtype selective antagonists [9]. These training sets were compiled from published affinity data (K_i_ values from competition assays using recombinant receptors expressed in cell lines) for as wide a range of structural classes of antagonists as was possible. For α_1A_ and α_1D_ pharmacophores only those compounds that exhibited >100-fold selectivity over α_1B_ and >40-fold selectivity over the other subtype (as calculated by the ratio of K_i_ values) were included. A set of conformations for each compound in each training set was also generated within the pharmacophore development program Catalyst. As described in our publication [9], Catalyst generates predictive pharmacophores by essentially a three dimensional pattern matching algorithm and extensive statistical analysis. The resulting pharmacophore hypotheses were extensively analysed and validated and finally reduced to one pharmacophore model for each of the three α_1_ –ARs. The α_1A_ pharmacophore consists of four features, describing a hydrogen bond acceptor (HBA), a hydrophobic aliphatic (Hal) and a hydrophobic aromatic group (Har), and a basic amine (PI). The training set for this pharmacophore included the two classes of antagonists as described by Klabunde and Evers [16], as well as structurally different compounds, which fitted into neither classification. The resultant pharmacophore was, however, weighted towards the class I antagonists, as they show higher selectivity and affinity than other antagonists. The α_1B_ pharmacophore was generated from antagonists showing selectivity over the other two subtypes, which were predominantly prazosin analogues. This pharmacophore did not include a basic amine feature, as may have been expected, but included 2HBA, Har and Hal features. A five-feature pharmacophore was generated for α_1D_ antagonists. The pharmacophore consisted of HBA, 2Hal, Har and PI features. The training set was heavily populated with analogues of BMY-7378, as these compounds were the only antagonists available which exhibit the appropriate type of selectivity. Despite this limitation of the training set, the pharmacophore was able to accurately predict the affinities of structurally distinct antagonists within a test set.

In this study, use of these pharmacophores in virtual screening of the National Cancer Institute and Tripos LeadQuest compound databases has identified 12 compounds with predicted high affinity and selectivity for the α_1D_-AR. 9 of the 12 isolated compounds displayed affinity at the α_1A_ and α_1D_ -AR subtypes, and all compounds with α_1_-AR affinity displayed negative efficacy. A lead compound with α_1A/D_-AR selectivity and a novel structural scaffold of an isoquinoline fused three-ring system was identified for future drug development projects.

## Materials and Methods

Compounds **1–6** were purchased from Tripos, Inc. (England) and compounds **7–12** were obtained via the Developmental Therapeutics Program of the National Cancer Institute (USA) (http://dtp.cancer.gov/). Drugs were solubilised in dimethyl sulfoxide (DMSO) at 10 mM and stored at −80°C. (-)-Epinephrine, (-)-norepinephrine, phentolamine hydrocholoride, lithium chloride (LiCl) and ±-propanolol were purchased from Sigma-Aldrich. [^3^H]Prazosin (85 Ci mmol^−1^) was from Perkin Elmer and *myo*-[^3^H]-Inositol (13 Ci mmol^−1^) was from Amersham. AG 1-X8 resin (100–200 mesh, formate form) was from Bio-Rad. Chemicals for buffered solutions (HEPES, EGTA and MgCl_2_) were obtained from Sigma-Aldrich. Other chemicals used were of the highest purity available.

### In Silico Screening

The chemical databases NCI2000 (238,819 compounds) and Tripos' LeadQuest Sample GPCR subset (3040 compounds) were searched in Catalyst version 4.11 (Accelrys Inc, San Diego, USA) using four and five feature subtype specific pharmacophores that we have previously developed [9]. The NCI2000 database was included with the Catalyst installation. The Tripos' LeadQuest Sample GPCR subset was acquired from JPRTechnologies as an .sd file. The .sd file was imported into Catalyst. This process included generating a set of conformations for each molecule in the database. Each search was performed with the “Best flexible” search option with no maximum limit on the number of hits returned. This search option performs a flexible fit for each conformation of each molecule in the database against the pharmacophore. Unique hits for each subtype were determined using the Boolean operator ‘OR’ provided in Catalyst, for example the hitlists after searching the NCI2000 database with the three pharmacophores for α_1A_-, α_1B_- and α_1D_-ARs were compared to each other and only unique hits (hits found only in one of the three lists) were kept. Estimates of fit were determined individually for each compound using the ‘Rigid’ fit option after ‘Best’ conformational searching, with the number of features allowed to miss set to one.

### Cell Culture and Transient Transfection

COS-1 cells (American Type Culture Collection, Manassas, VA) were cultured in Dulbecco's modified Eagle's medium (DMEM) supplemented with 10% (v/v) heat inactivated fetal bovine serum (FBS), 100 µg ml^−1^ penicillin and 100 µg ml^−1^ streptomycin. Cells were maintained and passaged upon reaching confluence by standard cell culture techniques. pMT4 plasmids containing rodent α_1_-AR (a kind gift from Prof R Graham, Victor Chang Cardiac Research Institute, Australia) were used to transfect cells. Transient expression in COS-1 cells was accomplished using two techniques; the diethylaminoethane-dextran (DEAE-dextran) and Lipofectamine 2000 (Invitrogen, Carlsbad, CA, USA) methods. Briefly, the DEAE-dextran method involved washing of cells with phosphate buffered saline (PBS), followed by the addition of the DNA mixture (12–25 µg/4×10^6^ cells) in DEAE-dextran (10 mg ml^−1^) to cells at 60–80% confluency. Following a 3 h incubation, a PBS wash with 10% DMSO was used to shock the cells. Cells were harvested 72 h post transfection. For the Lipofectamine 2000 method, cells were incubated for 12 h with Opti-MEM (GIBCO BRL, Gaithersburg, MD, USA) containing Lipofectamine 2000.

### Membrane Preparation

Membranes were prepared as described previously [17]. Briefly, COS-1 cells were transiently transfected using the DEAE-dextran method. Cells were homogenized with a Dounce homogenizer and nuclear debris was removed by centrifugation at 1260× g for 5 min. The membranes were resuspended in HEM buffer (20 mM HEPES, pH 7.4, 1.4 mM EGTA, and 12.5 mM MgCl_2_) containing 10% (v/v) glycerol and stored at −80°C until use. Protein concentration was determined using Bradford reagent (Sigma, St Louis, MO, USA).

### Ligand Binding Assay

Competition binding reactions contained HEM buffer, 200 pM of [^3^H]Prazosin, COS-1 membranes, and increasing amounts of unlabelled National Cancer Institute or Tripos LeadQuest compounds in a total volume of 200 µL. In saturation binding experiments, membranes in HEM buffer were incubated with various concentrations of [^3^H]Prazosin in a total volume of 200 µL. For all binding experiments, the reaction mixture was incubated for 1 h at room temperature, stopped by the addition of 4°C PBS and filtered with a Brandel cell harvester (Gaithersburg, MD, USA) on GF/C filters. Nonspecific binding was defined as binding in the presence of 100 µM phentolamine hydrochloride.

### Phosphatidylinositol Hydrolysis Assay

Accumulation of [^3^H] inositol phosphates (IPs) was determined as described previously [18]. Briefly, COS-1 cells were transiently transfected with α_1A_, α_1B_ and α_1D_ - AR pMT4 plasmids using the Lipofectamine method. 24 h after transfection, cells were seeded into 96 well plates and labelled overnight with 20 µCi ml^−1^ [^3^H]myo-inositol in inositol free DMEM supplemented with 5% charcoal stripped FBS. Cells were washed twice with inositol free DMEM, and incubated in inositol free DMEM for 2 h at 37°C. Cells were treated for 20 minutes with LiCl (20 mM) and propanolol (20 µM) in the presence or absence of tested compounds. Agonists were then added for 30 min, and the reaction was terminated by addition of 0.4 M formic acid. Cells were lysed by freeze thawing twice and were applied to AG 1-X8 columns. Total IPs were eluted with 1 M ammonium formate in 0.1 M formic acid. 200 µL of eluted sample was diluted with 1 mL reverse osmosis water and 5 mL Ultimaflow scintillation fluid (Perkin Elmer), and were counted in a liquid scintillation counter.

### Data analysis

Nonlinear regression analysis of saturation and competition binding assay data was performed using the noniterative curve fitting program GraphPad Prism (San Diego, CA, USA). Inhibition constants (*K*_i_) were determined by transformation of the program-calculated IC_50_ (concentration of ligand resulting in 50% inhibition of [^3^H]prazosin) value using the Cheng-Prusoff equation. The competitive binding data for each ligand was tested for both one and two -site binding. Subsequently a one-site binding model was determined as the appropriate form of analysis for all binding data. Receptor densities (B_max_) and the dissociation constant (*K_D_*) for [^3^H]-prazosin were calculated using the specific binding of the radioligand. Statistically significant differences (p<0.05) in the affinities of compounds were determined using one way ANOVA and Student-Newman-Keuls multiple comparison test.

## Results

### Database screening

The published α_1_ AR pharmacophores for subtype selective antagonists were used to search the NCI2000 and the aminergic GPCR subset of Tripos' LeadQuest databases in Catalyst. The NCI2000 database contains 238,819 compounds, and the LeadQuest GPCRDB has 3040 compounds. In each case the entire database was searched with each subtype's pharmacophore using the “best flexible” search option.

Searching the NCI2000 database returned 7630 hits for α_1A_, 49386 hits for α_1B_ and 3746 hits for α_1D_. In order to extract compounds that were returned as hits by only one of the three pharmacophores (unique hits), Boolean operators were used in Catalyst. Using this method it was determined that there were 1866 unique α_1A_ hits (0.78% of the whole database), 37746 unique α_1B_ hits (15.81%) and 446 unique α_1D_ hits (0.19%). It is interesting that substantially more hits were returned by the α_1B_ pharmacophore than by α_1A_ and α_1D_. This may well be due to the ‘positive ionisable’ (PI) features of the α_1A_ and α_1D_ pharmacophores being more restrictive in terms of database searching. The PI feature in Catalyst is used to describe an atom which is expected to be positively charged at physiological pH. In the case of ligands at adrenergic receptors, this is usually a basic nitrogen atom.

All unique hits were fitted to the pharmacophores to estimate their affinities at each α_1_-AR subtype. The α_1A_-AR pharmacophore returned 11 compounds with predicted picomolar activity. Many of the top compounds exhibited three fused rings, one compound (NCI0025463) had six fused rings. The α_1B_ pharmacophore returned 25 hits with predicted affinities in the picomolar range. Many of the returned hits were structurally similar to cyclazosin, a known α_1B_ selective ligand, displaying fused rings with substituents on analogous ring atoms. The α_1D_ pharmacophore returned a number of low nanomolar unique hitters with a variety of structures. Compound NCI0009677 is an adrenaline analogue with two long carbon chains. Two hits (NCI0169489 and NCI0167773) have a fused ring system similar to the compounds boldine and IQC, which have been previously investigated for their activity at the ARs [19].

The GPCR subset of the Tripos LeadQuest database contains compounds which fit a general pharmacophore of a basic nitrogen and an aromatic centre. Our pharmacophore screen returned 1125 hits for α_1A_-AR, 2144 hits for α_1B_-AR and 944 hits for α_1D_-AR. These hit-lists contained 157 unique α_1A_-AR hits (5.16%), 732 unique α_1B_-AR hits (24.1%) and 97 unique α_1D_-AR hits (3.19%). Of these unique hits 33 of the α_1A_-AR, 5 of the α_1B_-AR and 1 of the α_1D_-AR hits had picomolar predicted affinities. The top hits for α_1A_-AR were all relatively linear structures and most contained aromatic rings at both ends of the structure and a piperazinyl, or similar, central ring. The α_1B_-AR hits were similar, but had more branching evident in the central parts of the structures. The best α_1D_-AR hits exhibited a similar structural layout to the α_1A_-AR hits, but in general were shorter in length.

**Figure 1:**
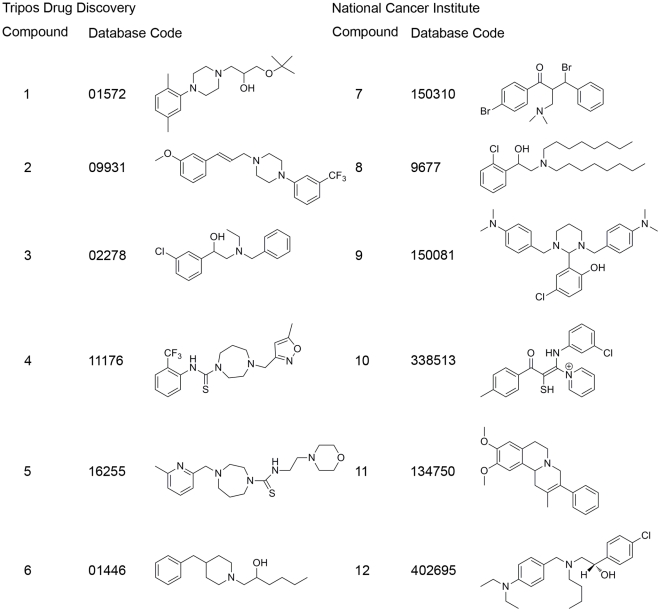
Compound numbers, database codes and structures of selected hits. Compounds were selected based on diversity and novelty of structure, predicted affinity and selectivity for the α_1D_-AR as well as availability from the Tripos LeadQuest aminergic GPCR and National Cancer Institute 2000 database. The stereochemistry of compounds **1, 2, 3, 6, 7, 8, 10** and **11** is not known.

Six compounds were selected from each of the NCI and LeadQuest GPCR database unique α_1D_-AR hitlists for *in vitro* testing at the α_1_ ARs (Figure 1). The compound selection was based on diversity and novelty of structure (not related to structures found in the training sets, or known to have affinity at adrenergic receptors), predicted affinity and selectivity for the α_1D_-AR as well as availability.

### Evaluation of compounds at cloned α_1_- adrenoceptor subtypes

The selected compounds (Figure 1) were assessed for ligand binding characteristics on membrane-expressed α_1_-ARs ([^3^H]Prazosin K_D_: α_1A_ 275±59; α_1B_ 200±46; α_1D_ 153±33 pM, n = 3–4). All competition radioligand binding curves displayed sigmoidal binding with the Hill slope for all experiments not significantly different from 1.0 (p<0.05), suggesting all compounds compete at one site with [^3^H]-Prazosin.

With the exception of compounds **4**, **5** and **10**, all compounds displayed affinity for the α_1_-ARs in the micromolar range (Table 1). Moderate affinity (*K*_i_<2 µM) was exhibited by compound **1** at all α_1_-AR subtypes, with compound **11** also displaying moderate affinity at the α_1A_- and α_1D_- ARs. Compound **2** displayed the highest affinity of all compounds with a *K*_i_ of 0.49 µM at the α_1A_-AR. Six of the nine compounds which had α_1_-AR affinity exhibited selectivity for α_1A_-AR and/or α_1D_-AR over α_1B_-AR (Table 1). The greatest selectivity was displayed by **2** at the α_1A_-AR, being 10- and 38-fold more selective over the α_1B_- and α_1D_- respectively (p<0.0005). Increased selectivity for α_1A_ (7-fold) and α_1D_ (17-fold) compared to α_1B_ (p<0.005) was observed with compound **11**.

**Table 1:** Binding affinities for selected pharmacophore hit compounds at the α_1_-adrenoceptors (ARs).

	α_1A_ –AR			α_1B_ -AR			α_1D_ -AR		
Compound	p*K*_i_ a	*K*_i_ b (µM)	n	p*K*_i_ a	*K*_i_ b (µM)	n	p*K*_i_ a	*K*_i_ b (µM)	n
**1**	5.93±0.02^D^	1.17	4	5.85±0.02^D^	1.42	3	6.10±0.07	0.83	4
**2**	6.31±0.03	0.49	4	5.34±0.04^A^	4.59	4	4.98±0.24^A^	18.8	5
**3**	4.90±0.15	15.1	4	5.02±0.51	24.4	4	5.11±0.09	8.35	4
**4**	Ø	Ø	3	Ø	Ø	3	Ø	Ø	3
**5**	Ø	Ø	3	Ø	Ø	3	Ø	Ø	3
**6**	5.31±0.08	5.22	4	4.87±0.20	17.0	4	5.26±0.10	5.90	4
**7**	4.83±0.24	20.3	3	4.77±0.39	38.5	3	5.01±0.29	59.0	3
**8**	4.38±0.13	45.9	3	Ø	Ø	3	4.31±0.04	48.7	3
**9**	4.63±0.12	24.5	3	Ø	Ø	3	4.61±0.26	36.1	3
**10**	Ø	Ø	3	Ø	Ø	3	Ø	Ø	3
**11**	5.75±0.14^B^	2.00	3	4.96±0.12^D^	11.8	3	5.82±0.1	1.60	3
**12**	4.51±0.30	45.5	3	Ø	Ø	3	4.49±0.35	62.4	3

*n* represents the number of experiments, each performed in triplicate.

Ø represents no binding at concentrations up to 100 µM.

a p*K*_i_ (−Log *K*_i_).

b *K*_i_ (inhibition constants) are the antilog of mean p*K*_i_.

*K*_i_ values were calculated according to the equation of Cheng and Prusoff . *K*_i_ =  IC_50_/1 + ([L]/*K*_D_) when [L] is the radioligand concentration and *K_D_* its dissociation constant.

A indicates significant differences from the α_1A_ –AR (*p*<0.05).

B indicates significant differences from the α_1B_ –AR (*p*<0.05).

D indicates significant differences from the α_1D_ –AR (*p*<0.05).

### Evaluation of the efficacy of compounds at the α_1_- adrenoceptor subtypes

Those compounds displaying α_1_-AR affinity (*K*_i_) at concentrations less than 100 µM were subsequently screened to determine their efficacy at all α_1_-AR subtypes using a phosphatidylinositol hydrolysis assay. Compounds were administered alone (100 µM) or prior to the addition of 10 µM (-)-epinephrine, to screen for agonist or antagonist activity respectively. This dosage of (-) epinephrine was chosen as it resulted in a sub-maximal inositol phosphate (IP) response.

Across all α_1_-AR subtypes, treatment with compounds alone did not result in changes in IP production compared to that of the control (untreated COS-1 cells expressing the α_1_-AR subtype) (p>0.05, n = 3), with the exception of **8**, for which a distinct trend of increased IP production was observed at the α_1A_- and α_1D_-ARs.

Four compounds (**1**, **2**, **8** and **11**) were selected for further biological testing with the aim of confirming and characterizing their activity quantitatively in terms of IC_50_ (antagonists) or EC_50_ (agonist) values for the α_1A_-AR. Criterion for selection of a compound was either an affinity (*K*_i_) of ≤5 µM for the α_1A_-AR or agonist activity.

**Figure 2:**
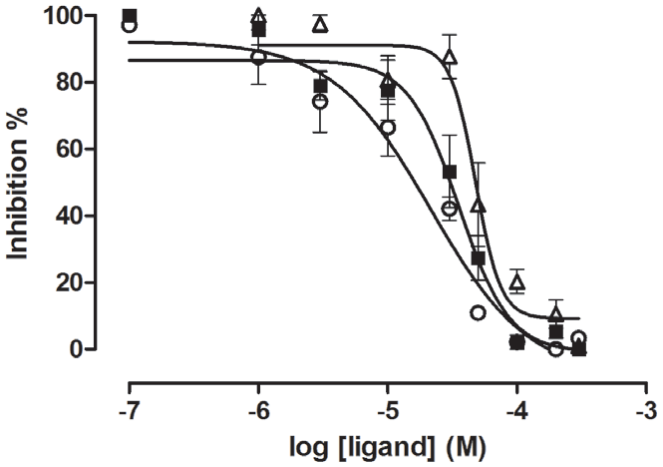
Inhibition of norepinephrine induced inositol phosphate (IP) accumulation at α_1A_- AR. α_1A_-AR transfected cells labelled overnight with [^3^H]- myo-inositol were treated with indicated concentrations (300 µM – 0.1 µM) of test compounds, **1** (▪), **2** (▵), and **11** (○), for 20 min. This was followed by a 30 min. stimulation with 10 µM (-)- norepinephrine, following which total IP accumulation was determined. Data was normalized against basal COS-1 cell IP production and expressed as a percentage of the (-)-norepinephrine-stimulated maximal IP response of that receptor subtype. The data represented the mean ± SE of a single experiment (n = 4–5), performed in triplicate.

Compounds **1**, **2**, and **11** concentration-dependently induced a decrease in intracellular IP accumulation following (-) norepinephrine stimulation at the respective α_1_-AR subtypes (Figure 2). All three antagonists have a similar potency at the α_1A_-AR (pIC_50_ (M) **11**, 4.88±0.27, n = 5, **1**, 4.60±0.11, n = 5 and **2**, 4.77±0.227, n = 4).

**Figure 3:**
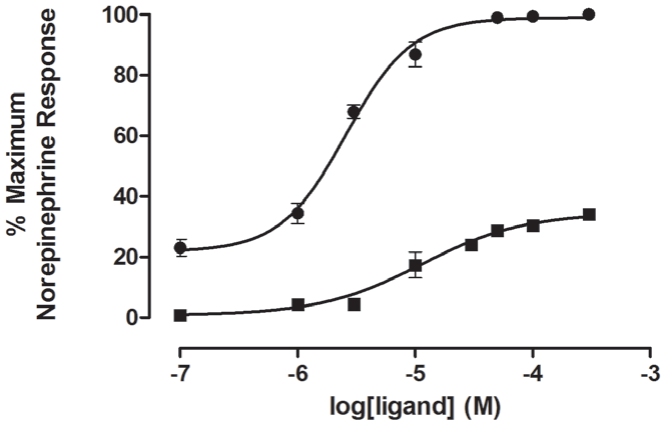
Inositol phosphate (IP) accumulation in the presence of norepinephrine and compound 8 at the α_1A_- adrenoceptor (AR). α_1A_-AR transfected cells were labelled with [^3^H]- *myo*-inositol. Following a 20 min. treatment with lithium chloride (20 mM) and propanolol (20 µM) the cells were stimulated with the indicated concentrations (300 µM − 0.1 µM) of (-) norepinephrine (•) or compound **8** (▪) for 30 min. Total inositol phosphate (IP) accumulation was determined as described under “Materials and Methods”. Data was normalized against basal COS-1 cell IP production and expressed as a percentage of the (-)-norepinephrine-stimulated maximal IP response. The data represents the mean ± SE of three separate experiments (n = 3), performed in duplicate or triplicate.

A dose dependent increase in intracellular IP was observed following treatment with compound **8** of COS-1 cells expressing the α_1A_-AR (pEC_50_, 5.26 ± 0.15 M (n = 3)). Maximal IP production induced by compound **8** was approximately 30% of the (-) norepinephrine control (Figure 3), indicating that this compound is a partial agonist.

### Mapping of compound 11 onto the α_1_-adrenoceptor pharmacophores

**Figure 4:**
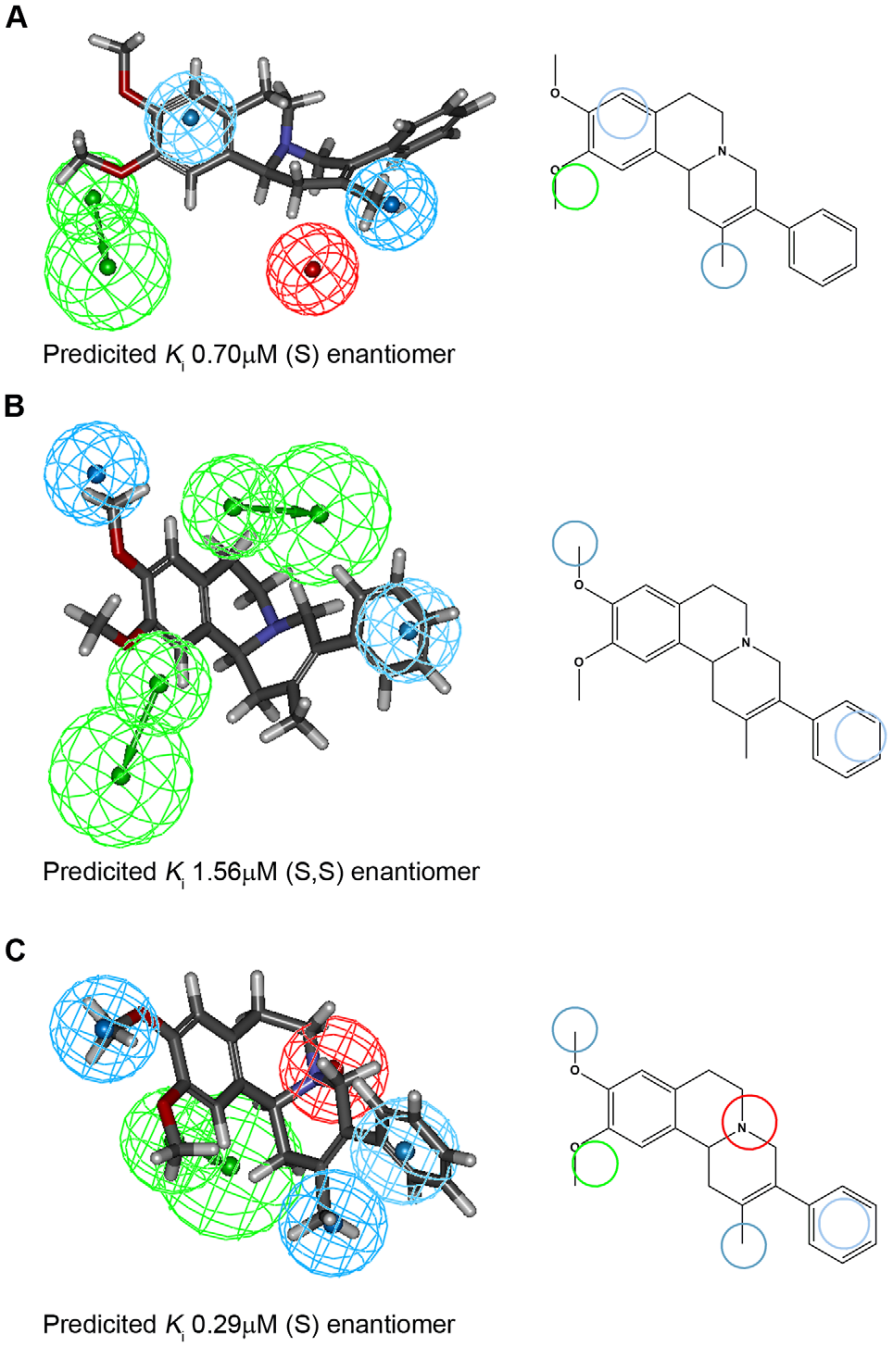
Mapping of compound 11 onto the α_1_-AR subtype pharmacophores. Pharmacophores for subtype selective antagonists for α_1A_-AR (**A**), α_1B_-AR (**B**), and α_1D_-AR (**C**) with compound **11** fitted onto them are shown. Three-dimensional (left) and two-dimensional (right) representations are shown for clarity. The pharmacophore features are represented by colour-coded spheres (red: positive ionisable; green: hydrogen bond acceptor; very light blue: hydrophobic aromatic; light blue: hydrophobic). These spheres depict location constraints where the feature should be situated on the molecule. For hydrogen bond acceptors, the proposed location of the interacting group on the α_1_-AR is also indicated by a second, larger, green sphere. Only features that are in the correct location (‘mapped’) are indicated in the two-dimensional images. The predicted binding affinity (*K*_i_) was determined as described in the methods section.

On the α_1A_ pharmacophore (Figure 4A), **11** only maps to three of the four features, missing the positive ionisable (PI) feature entirely (red sphere, top row) (Figure 4A). This feature represents an atom, such as a basic nitrogen, which is likely to be positively charged at physiological pH. The fit to the hydrogen bond acceptor feature (green) is poor. The poor fit of **11** onto the α_1A_-AR pharmacophore is consistent with the poor (micromolar) affinity of this compound at this receptor subtype, when compared to a typically nanomolar antagonist, such as the ones used in the development of the pharmacophore [9]. A similar situation is encountered for α_1B_ (Figure 4B), with only two of the four pharmacophore features mapped. Interestingly, when **11** is fitted onto the α_1D_ pharmacophore (Figure 4C), all five features are mapped, albeit with a poor fit for the hydrogen bond acceptor (green). This leads to the very high predicted affinity of **11** for the α_1D_-AR.

## Discussion

We have used pharmacophores to screen 241859 compounds *in silico*, from which 12 compounds were selected based on structural diversity and novelty, predicted high affinity and selectivity for the α_1D_-AR. 75% of the tested compounds display affinity below 100 µM at the α_1A_ and/or α_1D_ -AR subtypes and 50% at the α_1B_-AR. Furthermore, 25% of compounds have affinity in the low micromolar range (*K*_i_<5 µM) at the α_1A_-AR, and 17% at the α_1B_- and α_1D_-ARs. As these compounds only represent ‘hits’, it is not expected at this stage of the drug discovery process that compounds will have affinities comparable to established α_1_-AR ligands such as prazosin, which has a *K*_i_ value of approximately 0.1 nM at all α_1_-AR subtypes [20]. Indeed, a recent review of virtual screening by Klebe *et al.*, [21] found that of the around 50 targets addressed by virtual screening, most reported the discovery of ligands with micromolar binding affinities .

The ‘hit’ rate of 17–25% displayed by our α_1_-AR pharmacophores is comparable to the ‘hit’ rates achieved using the structure based virtual screening technique of docking at the serotonin 5-HT_1A_ and 5-HT_4_, dopamine D_2_, chemokine CCR_3_ and tachykinin NK_1_ receptors [22], for which ‘hit’ rates varied between 12–21% (*K*_i_ values <5 µM). A similar docking study using an α_1A_-AR homology model identified 37 novel antagonists, with an impressive ‘hit’ rate (*K*_i_ values <10 µM) of 46% [16]. Further, a pharmacophore screen by Aventis to identify antagonists for the urotensin II receptor showed a hit rate of 2% (*K*_i_ value cut-off not defined) [23].

The isolation of the α_1D_-AR selective **1**, α_1A_-AR selective **2**, and the α_1A/D_-AR selective compounds **8**, **9**, **11** and **12** indicates that the pharmacophores do contain structural motifs important for α_1_-AR subtype selectivity. Furthermore, as no selective α_1B_-AR compounds were isolated and 50% of the compounds were α_1A_ and/or α_1D_ –AR selective, this suggests screening the compound libraries using the α_1B_-AR pharmacophore did successfully identify and eliminate compounds with this characteristic from the pool of compounds.

The inability to distinguish between the α_1A_- and α_1D_-AR subtypes reflects the lack of α_1A/D_- AR selectivity of the training sets used to generate the α_1A_- and α_1D_-AR pharmacophores [9]. Compounds available at the time in the literature only exhibited >40-fold selectivity of α_1A_ over α_1D_-ARs, and therefore it is not expected that compounds isolated by the pharmacophores would display large α_1A/_α_1D_ selectivity.

α_1_-AR pharmacophores in the literature have been primarily used to screen the affinity of derivatives of established α_1_-AR ligands [24], [25], [26], [27], [28]. This approach involves the screening of compounds with narrow structural frameworks that are predetermined to have α_1_-AR affinity, with the purpose of isolating which chemical moieties provide the most advantageous characteristics of affinity and selectivity. In contrast to this, in this study large compound libraries of structurally diverse compounds have been screened, which has the benefit of identifying compounds with novel structural scaffolds. This represents the first attempt to screen diverse compound databases using α_1_-AR pharmacophores, representing a novel approach to the identification of α_1_-AR ligands.

There are currently eight established structural classes of α_1_-AR antagonists. All the compounds possess a central basic nitrogen, but it is the nature of the basic centre, the substitution of aromatic rings and the spatial orientation of chemical groups that determines subtype selectivity profile [29]. Compound **1** contains a piperazine ring motif, which is a common structural motif in α_1_-AR antagonists. There has been extensive research into this class of α_1_-AR antagonists, and analysis of the chemical structures of selective α_1_-AR antagonists indicates that a large group of active compounds contain the N-aryl or N-heteroaryl piperazine motif [30]. Accordingly, progress has been made in terms of understanding the chemical moieties of these derivatives important for α_1_-AR selectivity [28], [30], including significant details such as the size and positioning of substituents on the phenyl ring of the arylpiperazine motif [26], [31]. Despite this, selectivity against the α_2_-AR [26], [30] and the 5-HT_1A_ [28] receptors remain significant issues. Further, a study of more than 2000 compounds known to act at GPCRs that aimed to identify privileged substructures within GPCR ligands, identified the 4-phenyl-piperazine motif as the most frequently occurring, being present in 32 compounds which act at 13 different GPCRs [32]. Therefore, compound **1** does not represent a suitable lead compounds in terms of structurally novelty, and potential for subtype selectivity.

**Figure 5:**
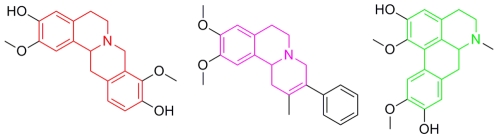
Fused ring systems as scaffolds for α_1_-AR antagonists. (**A**) Discretamine, with the 4-ring berbine motif highlighted in red. (**B**) Compound, **11**, with the novel fused isoqulinoline 3-ring motif highlighted in pink and (**C**) Boldine, with the 4 ring aporphine motif highlighted in green.”

Compound **11** however contains a novel fused isoquinoline three-ring system. Whilst fused four-ring motifs are found in berbine derivatives, such as the natural product (-) discretamine [33], and aporphine derivatives [34], both of which display α_1_-AR antagonism, a fused three-ring system has not previously been described for α_1_-AR ligands (Figure 5). Furthermore, the rigid core of compound **11** is an ideal template for developing a more bioactive flexible ligand [35]. Additionally, compound **11** contains few hydrogen bond forming groups and has a low molecular weight (335 Da), facilitating synthetic elaboration to enhance affinity and selectivity. While the free base has a relatively high calculated partition coefficient (clogP 4.9), salts can easily be prepared to enhance aqueous solubility.

We are currently developing a convergent synthesis of compound 11, allowing synthetic access to the molecule itself, as well as analogues for optimisation. In the absence of crystal structures of the three α_1_-AR subtypes, optimisation will be guided by docking into our previously described homology models [9] based on crystal structures of bovine rhodopsin as well as new models based on a number of new crystal structures of more closely related G-protein coupled receptors which have recently have been published [36].

In conclusion, the computer-aided drug discovery ligand-based pharmacophore technique has been established as a powerful alternative virtual screening tool. Compound **11** therefore represents a suitable lead compound for future drug development projects, due to its novel conformationally constrained structure, its “lead-like” properties and desirable affinity, efficacy and selectivity characteristics.
